# *N*-Methyl-2-thio­cytisine

**DOI:** 10.1107/S1600536810025870

**Published:** 2010-07-07

**Authors:** Anita M. Owczarzak, Anna K. Przybył, Maciej Kubicki

**Affiliations:** a Department of Chemistry, Adam Mickiewicz University, Grunwaldzka 6, 60-780 Poznań, Poland

## Abstract

The rings of the three-ring cytisine system in the title compound [systematic name: (1*R*,5*S*)-1,2,3,4,5,6-hexa­hydro-1,5-methano-8*H*-pyrido[1,2-*a*][1,5]diazo­cine-8-thione], C_12_H_16_N_2_S, have planar [maximum deviation  0.0170 (7) Å], half-chair and chair conformations. In the crystal structure, relatively short and directional C—H⋯π inter­actions and weaker secondary C—H⋯S contacts join the mol­ecules into helical chains along the [001] direction.

## Related literature

For general literature on cytisine, see: Okuda *et al.* (1961 ▶). For synthetic methods, see: Marriere *et al.* (2000 ▶); Imming *et al.* (2001 ▶). For similar structures, see: Freer *et al.* (1987 ▶); Imming *et al.* (2001 ▶). For asymmetry parameters, see: Duax & Norton (1975 ▶). For C—H⋯π interactions, see: Desiraju & Steiner (1999 ▶); Braga *et al.* (1998 ▶). For a description of the Cambridge Structural Database, see: Allen (2002 ▶). 
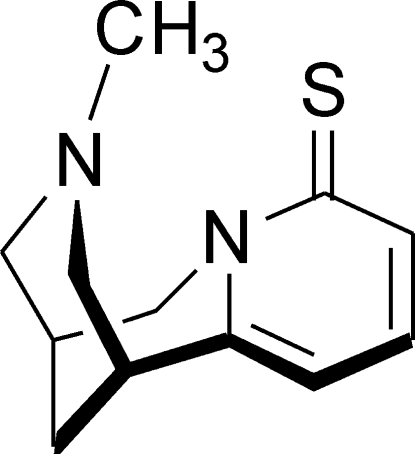


## Experimental

### 

#### Crystal data

C_12_H_16_N_2_S
*M* *_r_* = 220.33
Orthorhombic, 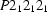

*a* = 9.8530 (6) Å
*b* = 10.6964 (7) Å
*c* = 10.8226 (7) Å
*V* = 1140.61 (13) Å^3^
*Z* = 4
Mo *K*α radiation
μ = 0.25 mm^−1^
*T* = 100 K
0.5 × 0.2 × 0.1 mm

#### Data collection

Xcalibur, Eos diffractometer
Absorption correction: multi-scan (*CrysAlis PRO*; Oxford Diffraction, 2009 ▶) *T*_min_ = 0.921, *T*_max_ = 1.000
13752 measured reflections
2744 independent reflections
2604 reflections with *I* > 2σ(*I*)
*R*_int_ = 0.019

#### Refinement

*R*[*F* ^2^ > 2σ(*F* ^2^)] = 0.021
*wR*(*F* ^2^) = 0.056
*S* = 1.07
2744 reflections
200 parameters
All H-atom parameters refined
Δρ_max_ = 0.25 e Å^−3^
Δρ_min_ = −0.16 e Å^−3^
Absolute structure: Flack (1983 ▶), 1015 Friedel pairs
Flack parameter: 0.02 (4)

### 

Data collection: *CrysAlis PRO* (Oxford Diffraction, 2009 ▶); cell refinement: *CrysAlis PRO*; data reduction: *CrysAlis PRO*; program(s) used to solve structure: *SIR92* (Altomare *et al.*, 1993 ▶); program(s) used to refine structure: *SHELXL97* (Sheldrick, 2008 ▶); molecular graphics: *Stereochemical Workstation Operation Manual* (Siemens, 1989 ▶); software used to prepare material for publication: *SHELXL97*.

## Supplementary Material

Crystal structure: contains datablocks I, global. DOI: 10.1107/S1600536810025870/nk2044sup1.cif

Structure factors: contains datablocks I. DOI: 10.1107/S1600536810025870/nk2044Isup2.hkl

Additional supplementary materials:  crystallographic information; 3D view; checkCIF report

## Figures and Tables

**Table 1:** Hydrogen-bond geometry (Å, °)

*D*—H⋯*A*	*D*—H	H⋯*A*	*D*⋯*A*	*D*—H⋯*A*
C5—H5⋯S2^i^	0.968 (15)	2.893 (14)	3.6861 (12)	139.9 (11)
C7—H7⋯S2^ii^	0.994 (14)	2.879 (14)	3.7338 (12)	144.6 (10)
C13—H13*A*⋯*Cg*1^ii^	0.983 (13)	2.648 (13)	3.5717 (14)	156.5 (10)

Symmetry codes: (i) 
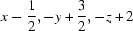
; (ii) 
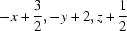
.

**Fig. 1.**
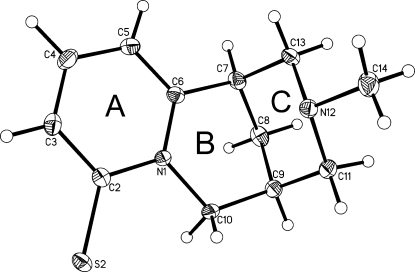
Anisotropic ellipsoid representation of molecule 1 together with atom labelling scheme. The ellipsoids are drawn at 50% probability level, hydrogen atoms are depicted as spheres with arbitrary radii.

**Fig. 2.**
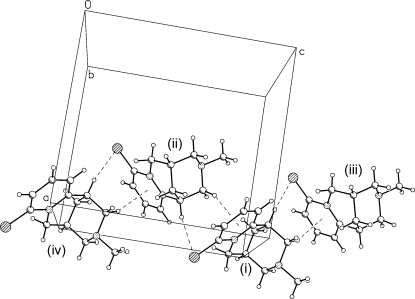
The chain of the molecules 1; weak hydrogen bonds are drawn as dashed lines. Symmetry codes: (i) x,y,z; (ii) 3/2 - x, 2 - y,-1/2 + z; (iii) 3/2 - x,2 - y,1/2 + z; (iv) x,y,-1 + z.

## supplementary crystallographic information

### Comment

(-)-Cytisine and its *N*-methyl derivative are toxic quinolizidine alkaloids, which are found in different plants from the *Fabaceae* (Leguminosae) family. They can cause nausea, convulsions and ultimately death by respiratory failure. Chemically, these compounds have a tricyclic skeleton, which can be characterized as a bispidine framework fused to 2-pyridone. The absolute configuration of two chiral centers was established as *7R*,*9S* (Okuda *et al.*, 1961), and the crystal structures of both cytisine and *N*-methylcytisine have been reported (Freer *et al.*, 1987). Also the structure of 2-thiocytisine was determined (Imming *et al.*, 2001), but this is the only structure of thioanalogue of cytisine in the CSD (Allen, 2002; Version 5.31 of Nov. 2009, updated Feb. 2010). During our studies on cytisine derivatives we have synthesized *N*-methyl-2-thiocytisine (1, Scheme 1), and here we present the results of its structural characterization.

The configuration *7R*,*9S* is confirmed by the value of the Flack parameter. The overall conformation of 1 is similar to other cytisine derivatives (Fig. 1). The ring A is almost planar, maximum deviation from the least-squares plane is 0.0170 (7) Å, ring B has the half-chair conformation, with five atoms almost coplanar (maximum deviation of 0.0497 (7) Å) and the bridgehead C8 atom significantly out of this plane, by -0.7560 (15) Å, and ring C is close to ideal chair conformation. This might be also described using the description of the asymmetry parameters (Duax & Norton, 1975), which measure the deviation from the ideal symmetry of the certain conformation. The ideal half-chair should possess *C_s_* symmetry, and the appropriate asymmetry parameter for B, ΔC_s_^N1^ is relatively high, equal to 8.4°. Ring C is much closer to the ideal chair symmetry of *D_3_**_d_*, maximum values of the asymmetry parameters are ΔC_s_^9^=2.61°, and ΔC_2_^8–9^=2.50°.

In the crystal structure - in absence of the possibility of stronger interactions - relatively short and directional, (Table 1) C—H···π interactions play quite an important role. Their geometrical characteristics fit quite well to the category of weak hydrogen bonds (*cf.* for instance Desiraju & Steiner, 1999, Braga *et al.*, 1998). Together with longer, probably of secondary nature, but still directional C—H···S contacts (Table 1) they connect the molecules, related by 2_1_ screw, into infinite chains along the [001] direction (Fig. 2).

### Experimental

(-)-Cytisine was isolated from the seeds of *Laburnum anagyroides* (Marriere *et al.*, 2000), white crystals, mp. 153 *^o^*C. *N*-methylcytisine was prepared adequately to the procedure: cytisine (1, 0.38 g, 2 mmol) was dissolved in 4 ml of 10% KOH and dimethyl sulfate (1.3 ml, 14 mmol) was added. The mixture was refluxed for 3 h. To cold mixture 12 ml of CH_2_Cl_2_ was added and the mixture was stirred for 20 min. Then organic layer was separated and washed with water, dried above MgSO_4_. The solvent was evaporated to give crude oil of *N*-methylcytisine that was purified on Al_2_O_3_. Yield 81%, (0.33 g) white crystals, m.p. 135–136 *^o^C*.

*N*-methyl-thiocytisine (1) was prepared according to the literature procedure of thiocytisine synthesis (Imming *et al.*, 2001). A mixture of *N*-methylcytisine (0.204 g, 1 mmol) and Lawesson's reagent (0.5 mmol, 0.202 g) was taken in a glass tube that was placed in an alumina bath inside the microwave oven and irradiated twice for 2 min. The crude material was dissolved in CH_2_Cl_2_ (40 ml) and filtered, the solvent was evaporated. The residue was purified by column chromatography (Al_2_O_3_, CH_2_Cl_2_). The yield 25% of yellow crystals (55 mg), m.p. 147 *^o^*C.

### Refinement

The positions of hydrogen atoms were found in the difference Fourier maps and both positional and isotropic displacement prameters were freely refined.

### Crystal data

**Table:**

C_12_H_16_N_2_S	*F*(000) = 472
*M**_r_* = 220.33	*D*_x_ = 1.283 Mg m^−^^3^
Orthorhombic, *P*2_1_2_1_2_1_	Mo *K*α radiation, λ = 0.71073 Å
Hall symbol: P 2ac 2ab	Cell parameters from 11220 reflections
*a* = 9.8530 (6) Å	θ = 3.4–29.0°
*b* = 10.6964 (7) Å	µ = 0.25 mm^−^^1^
*c* = 10.8226 (7) Å	*T* = 100 K
*V* = 1140.61 (13) Å^3^	Prism, colourless
*Z* = 4	0.5 × 0.2 × 0.1 mm

### Data collection

**Table:**

Xcalibur, Eos diffractometer	2744 independent reflections
Radiation source: Enhance (Mo) X-ray Source	2604 reflections with *I* > 2σ(*I*)
graphite	*R*_int_ = 0.019
Detector resolution: 16.1544 pixels mm^-1^	θ_max_ = 29.0°, θ_min_ = 3.4°
ω–scan	*h* = −12→12
Absorption correction: multi-scan (*CrysAlis PRO*; Oxford Diffraction, 2009)	*k* = −13→13
*T*_min_ = 0.921, *T*_max_ = 1.000	*l* = −14→14
13752 measured reflections	

### Refinement

**Table:**

Refinement on *F*^2^	Secondary atom site location: difference Fourier map
Least-squares matrix: full	Hydrogen site location: inferred from neighbouring sites
*R*[*F*^2^ > 2σ(*F*^2^)] = 0.021	H atoms treated by a mixture of independent and constrained refinement
*wR*(*F*^2^) = 0.056	*w* = 1/[σ^2^(*F*_o_^2^) + (0.040*P*)^2^] where *P* = (*F*_o_^2^ + 2*F*_c_^2^)/3
*S* = 1.07	(Δ/σ)_max_ = 0.001
2744 reflections	Δρ_max_ = 0.25 e Å^−^^3^
200 parameters	Δρ_min_ = −0.16 e Å^−^^3^
0 restraints	Absolute structure: Flack (1983), 1015 Friedel pairs
Primary atom site location: structure-invariant direct methods	Flack parameter: 0.02 (4)

### Special details

**Table:**

Experimental. For cytisine: EI—MS m/*z*: 190 (96%), m/*z*: 146 (100%), 147 (99%), 148 (42%), 134 (32%), 160 (29%), 109 (20%).For *N*-methylcytisine:EI—MS m/*z*: 204 (54%), m/*z*: 58 (100%), 146 (15%), 160 (9%). ^13^C-NMR: 163.5; 151.30; 138.5; 116.5; 104.6; 62.5; 62.1; 49.9; 46.2; 35.4; 35.4; 27.9; 25.4.For 1:EI—MS m/*z* 220 (92%), 162 (100%), 58 (47%), 189 (27), 176 (27%), 130 (23%)82 (17%), 117 (16%). ^13^C-NMR: 179.6; 133.5; 132.9; 154.4; 113.7; 62.6; 62.0; 58.4; 46.4; 36.3; 29.0. GC—MS analyses were performed on gas chromatograph CP3800 associated with mass spectrometer (4000MS, ion trap). The column was: VF-5 ms 30 m *x* 0.25 mm *x* 0.39 mm (Varian Part No. CP8944); carried gas was helium with flow 1 ml/min. Injector type 1177, std. on column, temp. 250 *^o^*C. Temperature program during GC—MS analysis: 80¯C/1¯C/1 min; 180¯C/20¯C/1 min/ 280¯C/10¯C/1 min, hold 20 minutes. NMR spectra were measured on a Bruker AVANCE 600 (600.31 MHz for 1H and 150.052 MHz for ^13^C) spectrometer.
Geometry. All s.u.'s (except the s.u. in the dihedral angle between two l.s. planes) are estimated using the full covariance matrix. The cell s.u.'s are taken into account individually in the estimation of s.u.'s in distances, angles and torsion angles; correlations between s.u.'s in cell parameters are only used when they are defined by crystal symmetry. An approximate (isotropic) treatment of cell s.u.'s is used for estimating s.u.'s involving l.s. planes.
Refinement. Refinement of *F*^2^ against ALL reflections. The weighted *R*-factor *wR* and goodness of fit *S* are based on *F*^2^, conventional *R*-factors *R* are based on *F*, with *F* set to zero for negative *F*^2^. The threshold expression of *F*^2^ > 2σ(*F*^2^) is used only for calculating *R*-factors(gt) *etc*. and is not relevant to the choice of reflections for refinement. *R*-factors based on *F*^2^ are statistically about twice as large as those based on *F*, and *R*- factors based on ALL data will be even larger.

### Fractional atomic coordinates and isotropic or equivalent isotropic displacement parameters (Å^2^)

**Table:**

	*x*	*y*	*z*	*U*_iso_*/*U*_eq_	
N1	0.90884 (9)	0.93342 (8)	0.93639 (8)	0.01107 (18)	
C2	0.93960 (11)	0.84480 (9)	0.84655 (9)	0.0124 (2)	
S2	1.05611 (3)	0.87330 (3)	0.73429 (2)	0.01503 (7)	
C3	0.86834 (12)	0.73023 (11)	0.85333 (10)	0.0161 (2)	
H3	0.8883 (14)	0.6684 (13)	0.7933 (13)	0.021 (3)*	
C4	0.77017 (12)	0.70978 (11)	0.94042 (11)	0.0195 (2)	
H4	0.7145 (14)	0.6306 (15)	0.9409 (13)	0.027 (4)*	
C5	0.74016 (12)	0.80336 (11)	1.02604 (10)	0.0174 (2)	
H5	0.6694 (15)	0.7997 (14)	1.0879 (12)	0.024 (4)*	
C6	0.80976 (11)	0.91409 (10)	1.02375 (9)	0.0129 (2)	
C7	0.77873 (12)	1.01441 (11)	1.11690 (10)	0.0150 (2)	
H7	0.6821 (15)	1.0028 (12)	1.1408 (12)	0.016 (3)*	
C8	0.80632 (12)	1.14397 (11)	1.06434 (10)	0.0174 (2)	
H8A	0.7864 (15)	1.2061 (15)	1.1303 (13)	0.026 (4)*	
H8B	0.7486 (14)	1.1589 (14)	0.9908 (12)	0.023 (4)*	
C9	0.95672 (12)	1.14670 (9)	1.03299 (10)	0.0150 (2)	
H9	0.9853 (14)	1.2232 (14)	0.9994 (12)	0.019 (3)*	
C10	0.98425 (12)	1.05431 (10)	0.92880 (10)	0.0137 (2)	
H10A	0.9562 (15)	1.0889 (13)	0.8476 (12)	0.020 (3)*	
H10B	1.0795 (16)	1.0292 (14)	0.9265 (13)	0.022 (4)*	
C11	1.04178 (12)	1.12303 (11)	1.14906 (9)	0.0160 (2)	
H11A	1.1421 (14)	1.1253 (14)	1.1292 (11)	0.018 (3)*	
H11B	1.0252 (14)	1.1959 (13)	1.2076 (12)	0.022 (4)*	
N12	1.00916 (10)	1.00157 (9)	1.20468 (8)	0.0139 (2)	
C13	0.86495 (12)	0.99465 (10)	1.23364 (10)	0.0154 (2)	
H13A	0.8353 (13)	1.0555 (12)	1.2962 (12)	0.015 (3)*	
H13B	0.8429 (13)	0.9123 (13)	1.2648 (12)	0.020 (3)*	
C14	1.08925 (14)	0.98412 (12)	1.31729 (11)	0.0202 (3)	
H14A	1.0719 (14)	0.9084 (15)	1.3549 (13)	0.025 (4)*	
H14B	1.1830 (16)	0.9908 (12)	1.2980 (12)	0.022 (4)*	
H14C	1.0686 (18)	1.0426 (17)	1.3806 (15)	0.039 (5)*	

### Atomic displacement parameters (Å^2^)

**Table:**

	*U*^11^	*U*^22^	*U*^33^	*U*^12^	*U*^13^	*U*^23^
N1	0.0113 (4)	0.0105 (4)	0.0114 (4)	−0.0004 (3)	0.0001 (3)	0.0008 (3)
C2	0.0118 (5)	0.0134 (5)	0.0120 (4)	0.0027 (4)	−0.0021 (4)	0.0009 (3)
S2	0.01402 (13)	0.01866 (13)	0.01242 (12)	0.00195 (11)	0.00257 (9)	−0.00147 (10)
C3	0.0192 (6)	0.0131 (5)	0.0161 (5)	0.0004 (4)	−0.0022 (4)	−0.0024 (4)
C4	0.0222 (6)	0.0154 (5)	0.0208 (6)	−0.0067 (5)	−0.0026 (5)	0.0016 (4)
C5	0.0170 (6)	0.0195 (6)	0.0157 (5)	−0.0047 (4)	0.0021 (4)	0.0011 (4)
C6	0.0112 (5)	0.0164 (5)	0.0112 (5)	0.0003 (4)	−0.0007 (4)	0.0015 (4)
C7	0.0126 (6)	0.0187 (5)	0.0138 (5)	0.0018 (4)	0.0029 (4)	−0.0026 (4)
C8	0.0202 (6)	0.0158 (6)	0.0162 (5)	0.0059 (5)	−0.0004 (4)	−0.0017 (4)
C9	0.0213 (6)	0.0092 (5)	0.0144 (5)	−0.0012 (4)	0.0009 (4)	0.0000 (4)
C10	0.0151 (6)	0.0120 (5)	0.0141 (5)	−0.0028 (4)	0.0025 (4)	0.0009 (4)
C11	0.0197 (5)	0.0131 (5)	0.0153 (5)	−0.0033 (5)	−0.0002 (4)	−0.0005 (4)
N12	0.0165 (5)	0.0126 (4)	0.0126 (4)	−0.0005 (3)	−0.0012 (3)	0.0012 (3)
C13	0.0182 (6)	0.0167 (5)	0.0112 (5)	−0.0007 (4)	0.0027 (4)	−0.0005 (4)
C14	0.0253 (7)	0.0179 (6)	0.0176 (6)	−0.0012 (5)	−0.0061 (5)	0.0016 (4)

### Geometric parameters (Å, °)

**Table:**

N1—C6	1.3747 (13)	C8—H8B	0.991 (14)
N1—C2	1.3913 (13)	C9—C10	1.5237 (15)
N1—C10	1.4936 (14)	C9—C11	1.5312 (14)
C2—C3	1.4143 (15)	C9—H9	0.939 (14)
C2—S2	1.6990 (11)	C10—H10A	0.993 (14)
C3—C4	1.3682 (16)	C10—H10B	0.976 (16)
C3—H3	0.948 (14)	C11—N12	1.4674 (14)
C4—C5	1.3957 (16)	C11—H11A	1.012 (13)
C4—H4	1.009 (16)	C11—H11B	1.018 (14)
C5—C6	1.3688 (16)	N12—C13	1.4570 (15)
C5—H5	0.968 (15)	N12—C14	1.4638 (15)
C6—C7	1.5037 (15)	C13—H13A	0.983 (13)
C7—C8	1.5225 (16)	C13—H13B	0.968 (14)
C7—C13	1.5371 (16)	C14—H14A	0.922 (16)
C7—H7	0.994 (14)	C14—H14B	0.950 (16)
C8—C9	1.5205 (16)	C14—H14C	0.950 (17)
C8—H8A	0.995 (15)		
			
C6—N1—C2	122.20 (9)	C10—C9—C11	113.73 (9)
C6—N1—C10	121.42 (9)	C8—C9—H9	113.3 (9)
C2—N1—C10	116.29 (9)	C10—C9—H9	103.0 (8)
N1—C2—C3	116.48 (9)	C11—C9—H9	107.3 (8)
N1—C2—S2	121.65 (8)	N1—C10—C9	115.61 (9)
C3—C2—S2	121.87 (8)	N1—C10—H10A	103.5 (8)
C4—C3—C2	121.69 (10)	C9—C10—H10A	111.3 (8)
C4—C3—H3	120.5 (8)	N1—C10—H10B	104.0 (9)
C2—C3—H3	117.8 (8)	C9—C10—H10B	111.6 (8)
C3—C4—C5	119.50 (11)	H10A—C10—H10B	110.3 (12)
C3—C4—H4	121.5 (8)	N12—C11—C9	111.29 (9)
C5—C4—H4	118.9 (8)	N12—C11—H11A	108.8 (8)
C6—C5—C4	120.16 (11)	C9—C11—H11A	110.8 (7)
C6—C5—H5	114.1 (9)	N12—C11—H11B	112.8 (8)
C4—C5—H5	125.7 (9)	C9—C11—H11B	107.2 (8)
C5—C6—N1	119.89 (10)	H11A—C11—H11B	105.7 (11)
C5—C6—C7	120.23 (10)	C13—N12—C14	109.89 (9)
N1—C6—C7	119.88 (9)	C13—N12—C11	110.29 (9)
C6—C7—C8	111.27 (9)	C14—N12—C11	109.66 (9)
C6—C7—C13	109.91 (9)	N12—C13—C7	110.81 (9)
C8—C7—C13	109.48 (9)	N12—C13—H13A	113.8 (8)
C6—C7—H7	106.3 (8)	C7—C13—H13A	108.1 (8)
C8—C7—H7	112.4 (8)	N12—C13—H13B	109.9 (8)
C13—C7—H7	107.3 (8)	C7—C13—H13B	106.7 (8)
C9—C8—C7	105.96 (9)	H13A—C13—H13B	107.2 (10)
C9—C8—H8A	109.9 (9)	N12—C14—H14A	112.3 (9)
C7—C8—H8A	107.7 (8)	N12—C14—H14B	109.4 (8)
C9—C8—H8B	112.1 (8)	H14A—C14—H14B	110.0 (12)
C7—C8—H8B	110.1 (9)	N12—C14—H14C	113.7 (10)
H8A—C8—H8B	110.8 (12)	H14A—C14—H14C	102.7 (13)
C8—C9—C10	109.04 (9)	H14B—C14—H14C	108.5 (13)
C8—C9—C11	110.31 (9)		
			
C6—N1—C2—C3	−3.20 (14)	N1—C6—C7—C13	−91.68 (11)
C10—N1—C2—C3	−179.95 (9)	C6—C7—C8—C9	−61.04 (12)
C6—N1—C2—S2	176.50 (8)	C13—C7—C8—C9	60.65 (11)
C10—N1—C2—S2	−0.25 (13)	C7—C8—C9—C10	65.86 (11)
N1—C2—C3—C4	2.75 (15)	C7—C8—C9—C11	−59.71 (11)
S2—C2—C3—C4	−176.95 (9)	C6—N1—C10—C9	7.35 (14)
C2—C3—C4—C5	−0.81 (17)	C2—N1—C10—C9	−175.87 (9)
C3—C4—C5—C6	−0.84 (18)	C8—C9—C10—N1	−39.82 (12)
C4—C5—C6—N1	0.42 (17)	C11—C9—C10—N1	83.75 (12)
C4—C5—C6—C7	−179.26 (11)	C8—C9—C11—N12	59.15 (12)
C2—N1—C6—C5	1.69 (15)	C10—C9—C11—N12	−63.71 (12)
C10—N1—C6—C5	178.28 (10)	C9—C11—N12—C13	−57.09 (12)
C2—N1—C6—C7	−178.62 (9)	C9—C11—N12—C14	−178.23 (9)
C10—N1—C6—C7	−2.04 (15)	C14—N12—C13—C7	179.28 (9)
C5—C6—C7—C8	−150.56 (10)	C11—N12—C13—C7	58.27 (12)
N1—C6—C7—C8	29.76 (14)	C6—C7—C13—N12	60.91 (12)
C5—C6—C7—C13	88.00 (13)	C8—C7—C13—N12	−61.59 (12)

### Hydrogen-bond geometry (Å, °)

**Table:**

*D*—H···*A*	*D*—H	H···*A*	*D*···*A*	*D*—H···*A*
C5—H5···S2^i^	0.968 (15)	2.893 (14)	3.6861 (12)	139.9 (11)
C7—H7···S2^ii^	0.994 (14)	2.879 (14)	3.7338 (12)	144.6 (10)
C13—H13A···Cg1^ii^	0.983 (13)	2.648 (13)	3.5717 (14)	156.5 (10)

Symmetry codes: (i) *x*−1/2, −*y*+3/2, −*z*+2; (ii) −*x*+3/2, −*y*+2, *z*+1/2.
